# Forcing external constraints on tree inference using ASTRAL

**DOI:** 10.1186/s12864-020-6607-z

**Published:** 2020-04-16

**Authors:** Maryam Rabiee, Siavash Mirarab

**Affiliations:** 1 Department of Computer Science and Engineering, UC San Diego, 9500 Gilman Dr, La Jolla, 92093 USA; 2 Department of Electrical and Computer Engineering, UC San Diego, 9500 Gilman Dr, La Jolla, 92093 USA

**Keywords:** ASTRAL, Constrained tree search, Tree completion, RF distance

## Abstract

**Background:**

To account for genome-wide discordance among gene trees, several widely-used methods seek to find a species tree with the minimum distance to input gene trees. To efficiently explore the large space of species trees, some of these methods, including ASTRAL, use dynamic programming (DP). The DP paradigm can restrict the search space, and thus, ASTRAL and similar methods use heuristic methods to define a restricted search space. However, arbitrary constraints provided by the user on the output tree cannot be trivially incorporated into such restrictions. The ability to infer trees that honor user-defined constraints is needed for many phylogenetic analyses, but no solution currently exists for constraining the output of ASTRAL.

**Results:**

We introduce methods that enable the ASTRAL dynamic programming to infer constrained trees in an effective and scalable manner. To do so, we adopt a recently developed tree completion algorithm and extend it to allow multifurcating input and output trees. In simulation studies, we show that the approach for honoring constraints is both effective and fast. On real data, we show that constrained searches can help interrogate branches not recovered in the optimal ASTRAL tree to reveal support for alternative hypotheses.

**Conclusions:**

The new algorithm is added ASTRAL to all user-provided constraints on the species tree.

## Background

Phylogeny inference requires solving an optimization problem over the space of all trees. The super-exponential growth of the tree topology space makes examining all trees impossible, even for moderately large datasets. As a result, tree inference algorithms have adopted several heuristics strategies, including iterative search (e.g., hill-climbing), used by most maximum parsimony and maximum likelihood methods.

An increasingly popular alternative is searching the tree space using dynamic programming (DP). For an optimization score of interest, we need a recursion formulating how the optimal tree on a subset of leaves (or similar constructs) can be computed from the optimal trees on smaller subsets. With a recursive equation, DP can be used to compute the optimal solution in the classic fashion, typically implemented using memoization. Since the powerset grows exponentially with the set cardinality, this DP requires exponential running time. However, a restricted version of DP can be designed where each set is divided into only some of its subsets; the restricted DP can have polynomial running time with respect to the number of the leaves.

Phylogenetic inference using this particular DP approach has been known at least as early as 1996 [1] and has been used for many optimization criteria, including duplication and loss [2–4], deep coalescence [5], Robinson Foulds (RF) distance [6], quartet score [7, 8], and others [9]. Among these, ASTRAL [8], which estimates a species tree from a set of gene trees by minimizing the quartet distance, has found increasing popularity [10]. DP is mostly used for problems where the input is a set of trees, and the output is a tree with the minimum total distance to the input trees. The popularity of DP for these problems is because the input set of trees create natural ways for restricting the space explored by DP. For example, the set of bipartitions observed in input trees can be used as the restriction set. In addition, ASTRAL has introduced heuristics to enrich the set of allowable bipartitions [11] while keeping the size of the search space polynomial [12].

The restrictions imposed on DP are not to be confused with the related concept of user-imposed constrained inference (we use “restricted” DP instead of “constrained” used in previous publications to avoid confusion). Systematists often would like to infer the best possible tree among trees that are compatible with a *constraint tree* of their choice, thereby completing and resolving the constraint tree. Constrained tree inference is needed for hypothesis-driven analyses that aim to choose the best among a set of hypotheses available by prior knowledge [13–16]. Constrained searches can help in model selection, testing whether a polytomy [17] or the monophyly of a group [18] can be rejected. Similarly, they can help gauge the “hidden” support for branches not recovered in the main analysis. Moreover, constrained searches have been successfully used to combine the results of multiple methods [19]. More recently, constrained trees were used in taxonomic profiling [20]. Finally, constrained searches enable updating existing trees without recomputing trees from scratch. For these reasons, most phylogenetic inference tools allow user-provided constraints.

To our knowledge, the DP paradigm has not been adopted to perform tree search with user-defined constraints. Performing constrained searches in the DP paradigm may appear easy: one needs to make sure the restricted set of bipartitions explored by DP are consistent with the constraints. As we show, there are roadblocks when the user-provided input is allowed to be arbitrary. The challenge is to find a large-enough search space that satisfies the user-provided constraints. Here, building on two recent advances [21, 22], we propose a solution to this challenge. We implement our solution inside the ASTRAL software for species tree inference, thereby enabling it to perform constrained searches for the first time. In extensive tests, we show that the constrained searches remain as accurate as unconstrained searches while reducing the running time, can improve accuracy in the presence of external knowledge about individual relationships, and can reveal hidden support.

## Method

Our goal is to extend ASTRAL so that it can honor a user-provided constraint tree. We start by reviewing ASTRAL and the RF(+) algorithm that we will use.

**Notations** We are given a set of *k* potentially multifurcating input trees $\mathcal {T}$ on subsets of a leafset $\mathcal {L}$ of size *n* and a potentially multifurcating constraint tree, $\bar {T}$ on a subset of $\mathcal {L}$. Let *l*(*t*) or *l*(*u*) be the set of leaves of a tree *t* or leaves below a node *u*. We use *s*(*u*) to denote sister(s) of *u*; i.e., the set of all nodes sharing a parent with *u*. Let $\mathcal {L}'=\mathcal {L}\setminus \{o\}$ where $o\in \mathcal {L}$ is a fixed arbitrarily chosen species. Denote $A\subset \mathcal {L}'$ as a cluster. Each edge in an unrooted tree corresponds to a bipartition of leaves, which corresponds to a cluster (the side missing *o*). A cluster *A* (i.e. the bipartition $A|\mathcal {L}\setminus A$) is called compatible with a tree *T* iff a tree exists that includes the bipartition and induces a resolution of *T* when restricted to same leaves as *T*. Two clusters are compatible iff they can be in the same tree [23].

### Background: tree completion

Completing a tree based on a reference tree is a well-studied problem and is often formulated as minimizing the distance to the reference tree while maintaining compatibility with the original tree [4, 21, 22, 24]. A natural objective is to find a complete tree with the minimum RF distance (i.e., the total number of bipartitions that differ between the two trees) to the reference tree [25]. OCTAL was the first quadratic time solution to this RF completion problem [21]. Bansal later introduced a linear time solution [22], which we call B-RF(+) algorithm. Both methods take as input an incomplete backbone tree, *T*_*b*_, and a complete and binary reference tree, *T*_*r*_, and output a binary and complete tree compatible with *T*_*b*_ such that the RF distance to *T*_*r*_ is minimized among all allowable trees.

**Fig. 1:**
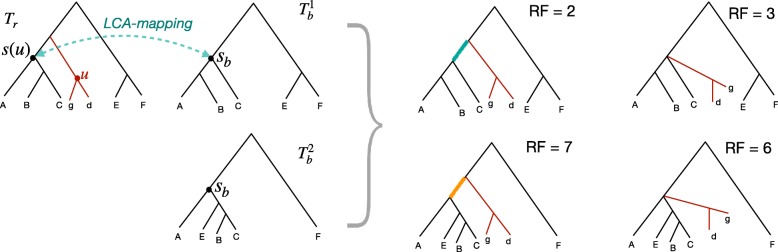
Updates need for the tree completion algorithm. $T_{b}^{1}$ and $T_{b}^{2}$ are both completed based on *T*_*r*_, generating either a binary tree or a multifurcating tree. In case 1 ($T_{b}^{1}$), subtree under *u* should be added as sister to *s*_*b*_ to minimize RF; the highlighted green branch matches *T*_*r*_, but creating a polytomy would result in a FN. In case 2 ($T_{b}^{2}$), the subtree under *u* should be added as a polytomy under *s*_*b*_; otherwise, the highlighted orange branch will be a FP. In the second case, restricting the output to be binary (as in the B-RF(+) algorithm) leads to suboptimal RF distances

The B-RF(+) algorithm [22] achieves linear time using constant time least-common-ancestor (LCA) lookups made possible after a linear time preprocessing using the Schieber–Vishkin technique [26]. Both trees are rooted on an arbitrary shared leaf. For every fully-missing node *u* of *T*_*r*_ (i.e., ∀*u*:*l*(*u*)∩*l*(*T*_*b*_)=*∅*), the subtree below *u* is added intact as the sister to the LCA of *l*(*s*(*u*)) inside *T*_*b*_ (Fig. 1). This placement of the subtree below *u* preserves all its bipartitions, the bipartition above it, and potentially the bipartition above the parent of *u* (we will come back to this point). The order of additions to *T*_*b*_ is determined by a pre-order traversal of *T*_*r*_, adding each fully-missing node *u* when we visit the parent node of *u*. Note that the topology of the backbone tree will not change by the addition of new subtrees. Bansal proved that this algorithm minimizes the RF distances between *T*_*r*_ and any possible *binary* tree that is compatible with *T*_*b*_.

### Background: DP algorithm implemented in ASTRAL

ASTRAL estimates an unrooted (species) tree given a set of unrooted (gene) trees $\mathcal {T}$ and is statistically consistent under the multi-species coalescent model [27] of incomplete lineage sorting (ILS) given a sample of true gene trees. ASTRAL seeks the tree *T* with the maximum quartet score to $\mathcal {T}$ defined as $\sum _{t\in \mathcal {T}} |Q(T)\cap Q(t)|$, where *Q*(.) is the set of quartet topologies of a tree. Let *S*(*A*) be the score for an optimal subtree on the cluster *A*. Defining *S*({*x*})=0 for $x\in \mathcal {L}$, the recursion is:

1 $$  {}S(A) \,=\, \max_{A' \in X, A \setminus A' \in X} \!S(A') + S(A \setminus A') + w_{\mathcal{T}}(A'\!|A\setminus A'\!|\mathcal{L} \!\setminus A)  $$

where *X* is a set of clusters and $w_{\mathcal {T}}$ is a function assigning weights to tripartitions of $\mathcal {L}$ such that the sum of all weights for any tree gives its quartet score. If *X* is set to $2^{\mathcal {L}'}$, the recursion tests all ways of dividing *A* into two smaller clusters and under this condition, $S(\mathcal {L}')$ (Eq. 1) gives the optimal quartet score [8] for $\mathcal {T}$ in time growing exponentially with *n* (expected, as the problem is NP-Hard [28]).

#### Forming set *X*: heuristics and restrictions

To handle large datasets, we need *X* to have a manageable size, preferably growing polynomially with *n* and *k*. At the same time, we ideally want *X* to have all clusters of the optimal tree. An obvious way of building *X* is to set it to all clusters in all trees in $\mathcal {T}$, hoping that all clusters in the optimal tree appear in at least one input. However, two difficulties emerge. Firstly, simulations under very high levels of gene tree discordance have shown this heuristic to be insufficient as biparti- tions in the optimal tree can frequently be absent from gene trees [11]. To deal with this issue, starting from ASTRAL-II, set *X* is enhanced using a set of heuristic methods, and since ASTRAL-III, the size of *X* is restricted to grow linearly with *n* and *k* [12, 29]. These heuristics (among other techniques) build consensus trees from input trees and add resolutions of polytomies of consensus trees to *X*.

The second difficulty is having full resolutions. Equation 1 is well-defined only if for every non-singleton *A*∈*X*, there is *A*^′^⊂*A* such that *A*^′^∈*X* and *A*∖*A*^′^∈*X*. More generally, a cluster *A* in *X* is useful *only if* there exists a fully binary tree on $\mathcal {L}'$ that includes *A* and all of its clusters are in *X*. Including any other cluster in *X* is a waste of computation. Thus, set *X* (which needs to be non-empty) needs to satisfy this property (recall $o\notin A\subset \mathcal {L}'$ and 2*n*−3 is the number of clusters in a fully resolved tree): P1: ∀*A*_1_∈*X*,∃{*A*_1_,*A*_2_,…,*A*_2*n*−3_}⊂*X* s.t.∀(*i*,*j*):*A*_*i*_ is compatible with *A*_*j*_.

Building *X* using bipartitions of input trees $\mathcal {T}$ can fail to satisfy this property unless all trees are complete and binary. Thus, starting from ASTRAL-II, three steps are taken. *i*) Before adding bipartitions from $\mathcal {T}$ to *X*, it first completes each tree with respect to other trees using a distance matrix computed from quartet frequencies in $\mathcal {T}$ and an algorithm based on the four-point condition [30]. *ii*) Polytomies in input trees are resolved once [11] or more [12] using heuristic methods that sample leaves around polytomies and use the distance matrix mentioned earlier. *iii*) Heuristic enhancements of set *X* employed in ASTRAL-II and ASTRAL-III are all explicitly designed such that P1 is automatically satisfied, a feat that has been particularly challenging for multi-individual datasets [29]. Thus, in effect, the set *X* includes all clusters from each tree in a set of binary and complete trees; the set includes modified input trees and others that ASTRAL heuristically selects.

### Enabling input constraints in ASTRAL

Given a constraint tree $\bar {T}$ and a set of gene trees, $\mathcal {T}$, our goal is to find the tree among all trees compatible with $\bar {T}$ that has the maximum quartet score with respect to $\mathcal {T}$. Compatibility with $\bar {T}$ is achieved if we enforce a second property on *X*. P2: ∀*A*∈*X*:*A* is compatible with $\bar {T}$.

Existing methods for forming *X* are not guaranteed to satisfy P2. One may think that we can follow standard methods of forming *X* and simply refuse to add clusters when they violate P2. Unfortunately, that approach, in addition to being slow, can violate property P1 and is not viable. Thus, the main challenge in building set *X* is maintaining P1 and P2 simultaneously and doing so in a scalable fashion.

#### Forming set *X* using tree completion

Our solution relies on completing and resolving the tree $\bar {T}$ using every tree in *X*. We require a tree completion method *Comp*(*T*_*b*_,*T*_*r*_) that adds to *T*_*b*_ leaves that are present in *T*_*r*_ but are absent from *T*_*b*_. The algorithm should only add missing leaves to *T*_*b*_ and can also resolve (some of) its polytomies. In other words, the output restricted to leaves of *T*_*b*_ is a resolution of *T*_*r*_; thus, $Comp(\bar {T},t)$ will be compatible with $\bar {T}$.

Tree completion/resolution methods were traditionally proposed for completing a gene tree using the species tree [4, 21, 22]. However, in our algorithm, we turn the problem on its head and complete the constraint species tree $\bar {T}$ using individual gene trees and use these mixed trees to build the set *X*. This uncommon use of the completion method is the main algorithmic idea that enables us to satisfy P1 and P2.

Given a *Comp*(*T*_*b*_,*T*_*r*_) method, we propose Algorithm 1 for forming set X. The first step is the primary new step and forces gene trees to be compatible with $\bar {T}$ (by definition of *Comp*(*T*_*b*_,*T*_*r*_)). Step 2 is identical to ASTRAL-III, where, compatible gene trees are completed in a reference-free fashion with respect to each other; any completion method such as the distance-based method used by ASTRAL [30] is valid here. Step 3, like ASTRAL-III, creates a set of multifurcating consensus trees ${\mathcal {C}}$ from the compatible gene trees $\bar {\mathcal {T}'}$. Then, in Step 4, like Step 2, we force consensus trees to be compatible with $\bar {T}$. Thus, all trees in $\bar {\mathcal {T}'}$ and $\bar {\mathcal {C}}$ are compatible with $\bar {T}$ and any resolution of these trees is also compatible with $\bar {T}$. Step 5 uses sampling methods of ASTRAL-III to resolve these trees heuristically and adds their bipartitions to *X*. Thus,


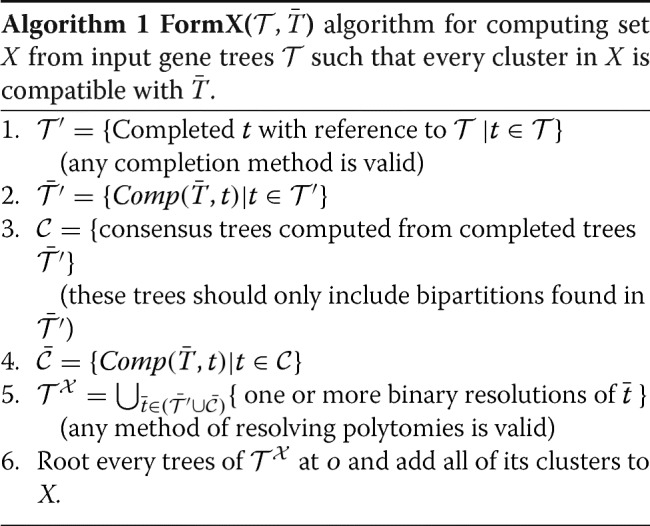


##### **Claim 1**

All bipartitions of *X* created by Algorithm 1 are part of a fully binary and complete tree (P1) and are compatible with the constraint tree $\bar {T}$ (P2).

After forming *X* (Algorithm 1), DP proceeds as before, computing $w_{\mathcal {T}}$ using the original gene trees $\mathcal {T}$. Since all bipartitions in *X* are compatible with $\bar {T}$, any tree formed by DP will be compatible with $\circ \bar{T}$; thus, no other changes are needed.

#### Tree completion with non-binary input/output

We now describe our choice for the *Comp*(*T*_*b*_,*T*_*r*_) method. We base our solution on the B-RF(+) algorithm [22] described earlier, which we re-implemented inside ASTRAL. However, several changes to the algorithm were necessary.

**Multifurcating output.** The B-RF(+) algorithm and OCTAL force the output to be binary. As a result, the output can include arbitrary branches that increase false positive (FP) edges (branches in the output missing from *T*_*r*_) without reducing false negative (FN) edges (branches in *T*_*r*_ missing from the output) (Fig. 1). Thus, if the output tree is allowed to be multifurcating, neither algorithm is optimal (shown by a counter-example; Fig. 1). As mentioned earlier, ASTRAL has several heuristics to resolve polytomies in the input trees (Step 5 of Algorithm 1), and these heuristics are preferable to an arbitrary resolution. Thus, we changed the B-RF(+) algorithm so that *Comp*(*T*_*b*_,*T*_*r*_) avoids adding arbitrary resolutions in Steps 2 and 4, leaving resolving polytomies to heuristics of Step 5. Our experiments show that this change substantially reduced the RF of completed trees (Fig. S1).

Recall that the B-RF(+) algorithm adds each fully-missing node *u* in *T*_*r*_ as sister to the LCA of *l*(*s*(*u*)) in *T*_*b*_; denote this LCA node as *s*_*b*_. Let ${\mathcal {L}^{m}} = l(T_{r})\setminus l(T_{b})$ be the set of leaves missing from *T*_*b*_ and let ${\mathcal {L}^{s}} = l(T_{r})\cap l(T_{b})$ be shared leaves. Two cases arise.

**Case 1.**$l(s(u))\setminus {\mathcal {L}^{m}} = l(s_{b})$. In this case, the optimal placement of the subtree below *u* in *T*_*b*_ is as sister to *s*_*b*_, creating a new node above *s*_*b*_. The reason is that this placement leads to the bipartition identified by *s*(*u*) to be identical between *T*_*r*_ and the completed tree, thereby avoiding a FN edge.

**Case 2.**$l(s(u))\setminus {\mathcal {L}^{m}} \neq l(s_{b})$. Here, no placement of the subtree below *u* onto *T*_*b*_ can avoid the FN penalty associated with missing the bipartition associated with *s*(*u*) in *T*_*b*_. However, placing the subtree as sister to *s*_*b*_ by creating a new internal node does lead to an unnecessary FP edge in the completed tree, separating *l*(*s*_*b*_) from other leaves (Fig. 1). To avoid these FP edges, we can simply create a polytomy in the completed tree by putting the new subtree as another child of *s*_*b*_.

Our new algorithm (called B-RF(*) here) is a straight-forward change of the B-RF(+) algorithm. We compute the LCA mapping both ways. When inserting the *u* subtree into *T*_*b*_ at *s*_*b*_, we check if the LCA of *s*_*b*_ in *T*_*r*_ matches *s*(*u*). If it does, we create a new internal node above *s*_*b*_; otherwise, we create a polytomy in *T*_*b*_ by adding the subtree as a child of *s*_*b*_. Let *T*^∗^ be the output of our updated algorithm and *T*^+^ be the output of the original B-RF(+) algorithm. By construction, every branch in *T*^∗^ is also present in *T*^+^, and thus, *T*^∗^ is a contraction of *T*^+^.

##### **Claim 2**

The output of B-RF(*) has the minimum RF distance to the binary tree *T*_*r*_ among all (potentially multifurcating) trees compatible with *T*_*b*_.

We prove Claim 2 by showing that *T*^∗^ has the minimum possible number of FN and FP branches; the optimality of RF follows. We rely on the result that *T*^+^ is optimal among all binary trees [22]. By optimality of *T*^+^, it has the minimum possible FN that any tree (binary or multifurcating) can achieve (a multifurcating tree cannot have a lower FN than *T*^+^ because binary resolution of that tree would also have a lower FN than *T*^+^). Also, *T*^∗^ is a contraction of *T*^+^, where, a branch is contracted *only if* it is a FP branch due to Case 2. Thus, the number of FN branches in *T*^∗^ equals those of *T*^+^ and hence is the minimum possible.

Now, let the number of FP branches in the *T*_*b*_ compared to $T_{r}\!\!\upharpoonright _{\mathcal {L}^{s}}$ be *f*. Since adding ${\mathcal {L}^{m}}$ to *T*_*b*_ cannot reduce its FP branches, *f* is a lower bound on the number of FP branches in the optimal tree. We claim that the number of FP branches in *T*^∗^ is also *f*. Starting with *T*_*b*_, every addition of a subtree *u* to the current tree (*T*_*i*_) keeps the number of FP branches fixed. To see this, first, consider a branch *b* of *T*_*i*_ that matches *T*_*r*_ (restricted to common leaves) before *u* is added and assume *u* is not placed on *b*; then, *b* cannot become a FP because *u* is placed on the correct side of *b* by the proof of the B-RF(+) algorithm. Next, consider the branch *b* where *u* is placed. If *b* matches *T*_*r*_ before the addition, *u* is placed here only if *l*(*s*(*u*)) matches leaves below *b*, as in Case 1, where two true positive branches are created. If *b* does not matches *T*_*r*_ before the addition, Case 2 ensures that we create a polytomy and avoid adding a new FP branch. Thus, every step keeps the number of FP branches fixed.

**Multifurcating backbone.** We defined B-RF(*) for binary *T*_*b*_, but we can have multifurcating *T*_*b*_ (here, $\bar {T}$). To adopt B-RF(*) to multifurcating backbones, prior to completion, we need to add to *T*_*b*_ those bipartitions in *T*_*r*_ that are compatible with *T*_*b*_ (or else we will have unnecessary FN branches). This can be done using the same LCA mapping of the B-RF(*) algorithm. In a post-order traversal of *T*_*r*_, for every node *u* that maps to a polytomy *v* in *T*_*b*_, we check whether all children of *u* have a LCA mapping to a child of *v*. If they do, we create a new node below *v* and move mapped children under *v* to be children of the new node. It is easy to see that this method adds all missing bipartitions from *T*_*r*_ to *T*_*b*_.

**Multifurcating reference.** Changing B-RF(*) to handle multifurcating *T*_*r*_ is straightforward. In the pre-order traversal, for any polytomy node *u* encountered in *T*_*r*_, when there are multiple fully-missing nodes under *u*, we add them as a group to the same position (as a polytomy) in *T*_*b*_. Other cases are naturally handled by the LCA mapping used by the B-RF(*) algorithm (note that we defined “sister” as the *set* of nodes sharing a parent with a node).

## Results: simulations

We first test constrained ASTRAL on an existing [11] simulated dataset with 201 species. This SimPhy [31] dataset has three model conditions with moderate, high, or very high levels of ILS, controlled by setting the maximum species tree height to 10^7^, 2×10^6^, or 5×10^5^ generations; mean RF distance (normalized by total number of branches in both trees) between the true species tree and true gene trees are 15%, 34%, and 69%, respectively. We use gene trees inferred using FastTree-II [32] from sequence data in our analysis. The estimated gene trees have relatively high levels of gene tree error (the average RF distance between estimated and true gene trees are 25%, 31%, and 47% for the three model conditions). Following previous publications [11], three replicates of high ILS dataset are removed due to a extreme lack of gene tree resolution.

We compare both constrained and unconstrained ASTRAL to the true tree. We measure the topological error using the normalized RF distance, and also report the change in quartet score and running time between constrained and unconstrained ASTRAL. Note that quartet score of the constrained tree can be higher than unconstrained ASTRAL, as the default version of ASTRAL has a heuristic definition of *X* and is not guaranteed to find the optimal solution. We ask whether our method of forming the constrained and restricted set *X* is effective in providing the same level of accuracy as unconstrained (but restricted) searches while improving the running time. We then ask if the use of constraints can benefit accuracy.

### Is set *X* restricted to a constraint tree sufficiently large?

#### Constraint tree $\bar {T}$ with missing leaves

We built constraint trees that include $\frac {1}{4}$, $\frac {1}{2}$, or $\frac {3}{4}$ of species by taking the ASTRAL-III tree on the full dataset and pruning leaves uniformly at random. Since the unconstrained tree induces the constraint tree, the relative accuracy of constrained and unconstrained search is entirely a function of the completeness of *X*.

**Fig. 2:**
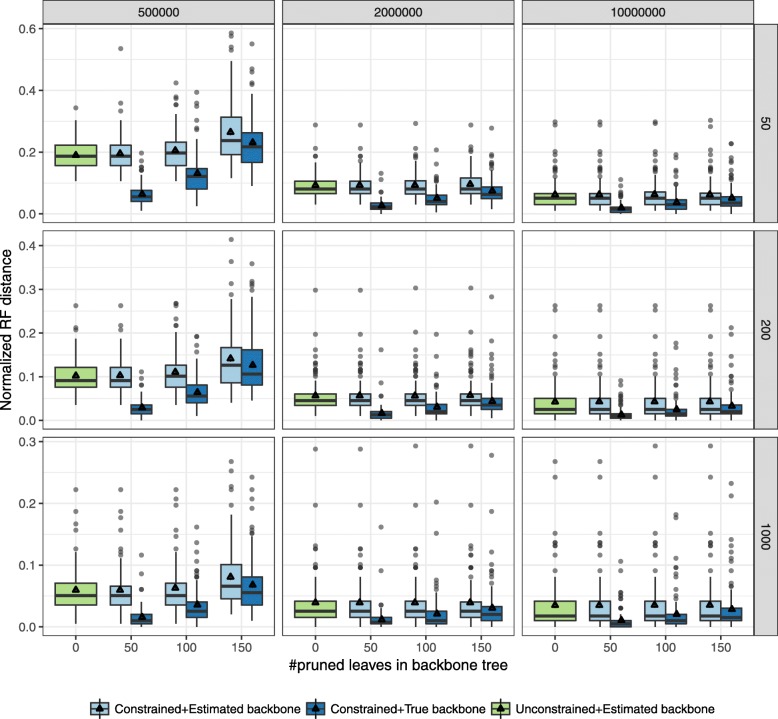
Effectiveness of constrained ASTRAL with constraint trees that have randomly distributed missing leaves. ASTRAL-III species trees are compared with and without constraints using the Normalized RF distance between inferred species tree and true species tree. Boxplots show distribution (over 100 replicates) and triangles show the mean. Panels correspond to three different levels of ILS (500K, 1M and 2M generations, corresponding to very high, high, and moderate ILS, respectively) and varying number of genes (50, 200, and 1000). The constraint trees are obtained by pruning 50, 100, or 150 (x-axis) randomly chosen leaves from the unconstrained ASTRAL tree or the true species tree

The accuracy of the constrained ASTRAL in most condition matches that of the unconstrained ASTRAL (Fig. 2). For moderate (10^7^) and high (2×10^6^) levels of ILS, the drop in average accuracy for different numbers of genes never exceeds 0.4*%*. Only with very high ILS (5×10^5^) and constraint trees with 50 species do we start to see small but noticeable drops in the accuracy of constrained ASTRAL. For example, with 50 genes and very high ILS, the error goes up from 20% with no constraints to 26% with a $\bar {T}$ that has 50 leaves. Consistent with this, the quartet score of the ASTRAL tree also remains largely unchanged in most cases, except, again, for the case of very high ILS and backbone trees that include only 50 leaves (Fig. S2).

**Fig. 3:**
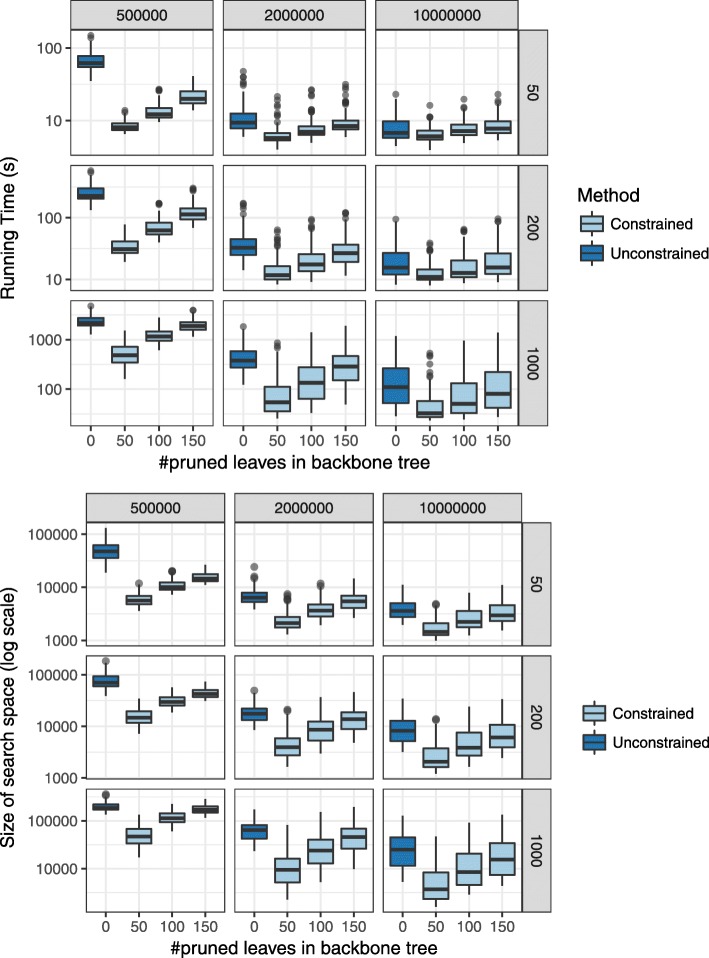
Impact of constrained search on the running time and search space. The running time (top) and search space size |*X*| (bottom) are compared between constrained and unconstrained ASTRAL-III. Other settings of the figure are identical to Fig. 2

Constrained searches also impact the running time and the search space *X* (Fig. 3). With very high ILS and $\bar {T}$ including 150 of leaves (pruning 50), we get from 4 to 8x improvement in running times and substantial reduction in the size of the search space. This reduction explains the small reduction in the accuracy of ASTRAL-III with constrained searches under these conditions. As backbone size becomes smaller, the running time converges to unconstrained ASTRAL; however, even when $\bar {T}$ includes a quarter of the leaves, we still have 1.2 to 3x improvement in the running time. With moderate and high levels of ILS, improvements are less pronounced but still substantial. Running time improvements mirror the change in the search space size, which is dramatically reduced (Fig. 3).

We also study a scenario where missing leaves in the constraint tree form clades instead of being uniformly distributed. Results of the clade-based removal did not substantially differ from the random removal (Fig. S3). With moderate or high ILS, random and clade-based removal were indistinguishable, and for very high ILS, only small differences were observed.

To summarize, our method of forming *X* for constraints with missing leaves retains accuracy and reduces running time, with only small reductions in the accuracy in the most extreme conditions, namely very high ILS and small constraint trees.

#### Constraint tree $\bar {T}$ with multifurcation

**Fig. 4:**
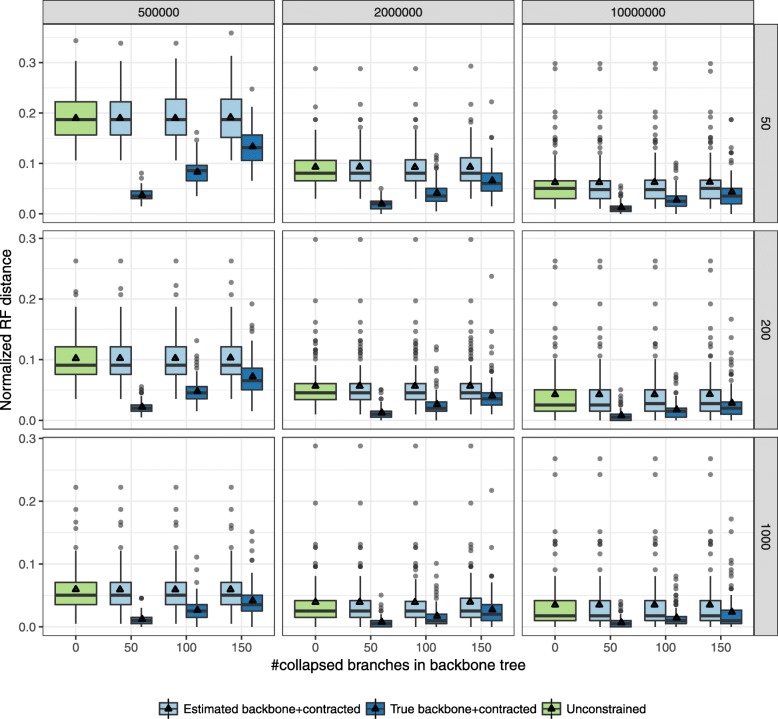
Effectiveness of constrained ASTRAL with constraint trees that have randomly distributed contracted branches. Settings are similar to Fig. 2. The constraint trees are obtained by collapsing 50, 100, or 150 (x-axis) randomly chosen branches from the unconstrained ASTRAL tree or the true species tree. Note that the set of branches contracted from the true and estimated species trees are not identical

We next collapse randomly chosen branches from the unconstrained ASTRAL-III tree to create a complete but unresolved constraint tree. With these multifurcating constraint trees, constrained ASTRAL search is as accurate as the unconstrained ASTRAL even for very high levels of ILS (Fig. 4). Differences in mean accuracy are no more than 0.1*%* in any of the 27 conditions we tested. Remarkably, in the case of very high ILS, we even see a small but noticeable improvement in quartet score (but not accuracy) when the constraint tree includes only 50 branches (Fig. S4). Once again, the running time and the size of the search space both reduce dramatically in the constrained searches (Figure S5). Thus, our method of forming *X* is effective in the face of multifurcating constraint trees.

### Can a constrained search help accuracy?

Constrained searches have the power to improve accuracy if prior knowledge of parts of the tree is available. To test this proposition in simulations, we study the accuracy of constrained ASTRAL when the constraint tree $\bar {T}$ is a subset or a contraction of the *true* species tree. In both cases (Figs. 2 and 4), the accuracy of the ASTRAL tree improves, and changes are dramatic when the constraint tree is missing only 50 leaves or branches. The improvements are especially strong for the case of complete but multifurcating true species trees (Fig. 4) where a constraint tree with only 50/198 branches can reduce the error from 19% to 13% with 50 genes with very high ILS. If $\bar {T}$ includes 150/198 branches, the error reduces down to 4%.

**Fig. 5:**
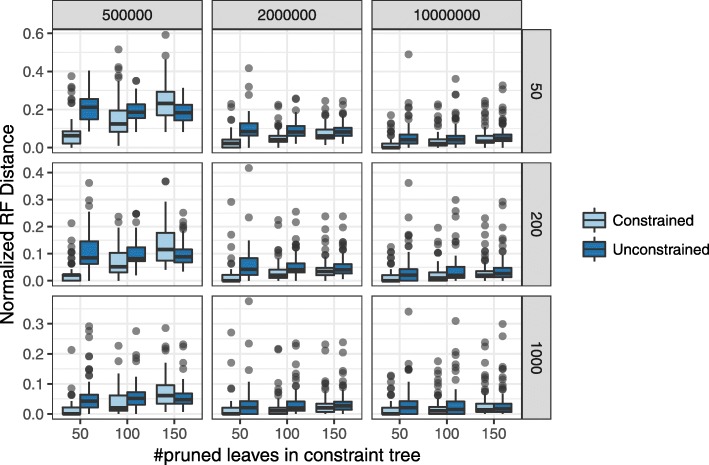
Impact of correct constraints on some branches on the remaining branches. We show the distribution of the RF distance between (constrained or unconstrained) ASTRAL trees and the true species tree when both trees are restricted to the set of leaves that are *not* present in the constraint tree. As in Fig. 2, constraint trees are defined by pruning 50, 100, or 150 leaves from the true tree

The dramatic improvements in accuracy are perhaps not surprising given the fact that parts of the tree are fixed to match the true tree. More interesting is whether adding constraints to some part of the tree improves the accuracy of the *remaining* parts of the tree. We thus evaluated the accuracy of trees only restricted to the leaves that are not part of the constraint tree. We observe that the accuracy of the remaining leaves has also increased dramatically as a result of having constraints (Fig. 5). For example, in the very high ILS case with 50 genes, when 50 species are missing from the correct constraint tree, the error for the placement of these 50 species has reduced from 21% with no constraints to only 7% with constraints. Similarly strong levels of improvement are observed across all conditions, except when the constraint tree includes only 50 leaves. To summarize, the result demonstrates that given correct prior knowledge about parts of the phylogeny, a constraint ASTRAL search can improve the accuracy of the remaining parts.

## Results: biological dataset

**Fig. 6:**
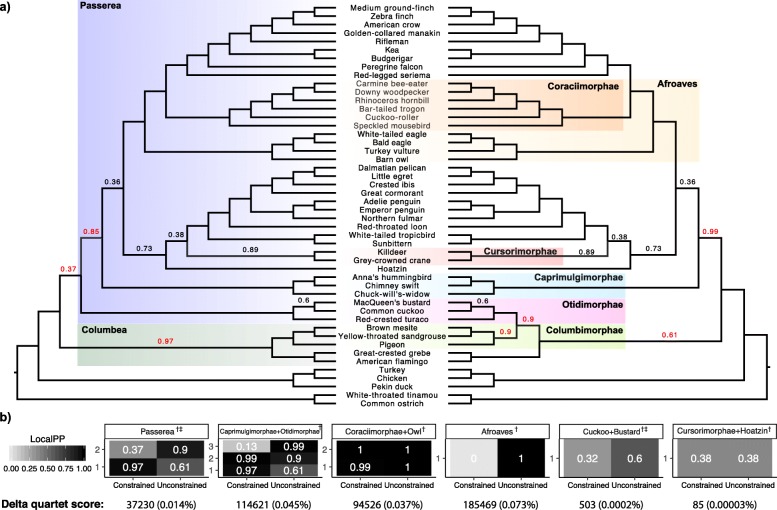
Constrained analyses reveal support for alternative clades. **a** On the avian genomics dataset [33], we estimated ASTRAL-III trees with no constraints (right) using 2022 binned (super)gene trees. The tree did not include the Passerea vs. Columbea division that most other analyses of this dataset reveal. Constraining the ASTRAL tree to include Passerea as a clade resulted in a tree (left) with two new branches and 0.97 localPP support for Columbea. LocalPP support values below 1.0 are shown on branches (red: change in support across the two trees). **b** Similar to (**a**), constrained analyses are performed to find support for five other clades found in the TENT (*†*) and (in some cases) in MP-EST* (*‡*) but not found in the unconstrained analyses. For each clade, we show localPP support for branches that differ between the constrained and unconstrained trees (one to three branches change in constrained searches). We also show the reduction in the quartet score in the constrained analyses as an abosulte number and percentage

We reanalyze the avian phylogenomic dataset [33] with 48 bird species and more than 14,000 loci. The statistical binning method has been proposed to enable coalescent-based analyses of this dataset despite the low phylogenetic signal [34]. The main novel result found using this dataset is the division to Passerea and Columbea at the base of Neoaves, a relationship that was recovered in most analyses of the dataset, including concatenation (TENT), MP-EST [35] run on binned gene trees (MP-EST*), and ASTRAL run on unbinned gene trees with low support branches contracted [12]. However, running ASTRAL on 2022 binned gene trees failed to recover Passerea and Columbea and placed Otidimorphae (a clade within Passerea) within Columbea (Fig. 6). Nevertheless, the localPP support [36] for this alternative relationship is low. Thus, using constrained searches, we now ask whether there is support for Columbea/Passerea in the binned gene trees.

Constraining the ASTRAL tree to include Passerea results in recovering Columbea and placing Otidimorphae as sister to other Passerea. The Columbea clade, absent from unconstrained ASTRAL, has high support (0.97 localPP) in the constrained tree. Moreover, the support of the Columbimorphae, a clade universally supported in modern analyses, increases from 0.9 in the unconstrained tree to 1.0. On the other hand, the localPP support for Passerea is only 0.37, which is barely above the expected support of a random resolution (0.33), and the total quartet score of the tree is reduced by 37230 quartets (0.015%). We then performed another constrained analysis forcing Otidimorphae to be with Caprimulgimorphae (as in TENT). This constraint leads to Passerea and Columbea both becoming monophyletic with 0.99 and 0.97 localPP (Fig. 6b). However, the Otidimorphae+Caprimulgimorphae clade itself has low localPP (0.13) and total quartet score reduces by 0.045%. Overall, while the unconstrained ASTRAL tree does not recover Columbea and Passerea, gene trees strongly support Columbea (if not Passerea).

We next tested four other clades recovered in TENT but absent from the unconstrained ASTRAL (Fig. 6b). Several patterns were observed. Forcing Afroaves to be monophyletic reveals a total lack of support for the monophyly of that clade (localPP =0 and 0.07% reduction in quartet score). Forcing Cuckoo to be sister to Bustard or Hoatzin to be sister to Cursorimorphae shows a case where neither the constrained nor the unconstrained tree have strong support, and thus, results are inconclusive. Most interestingly, owl fits quite well with Coraciimorphae (localPP 0.99 in constrained analyses) as well as its unconstrained position as sister to birds of prey (localPP 1.0); this observation creates a suspicion of gene tree discordance due to processes other than ILS such as hybridization.

## Discussion

The method we introduced for honoring user-provided constraints relies on an extension of a tree completion algorithm by Bansal [22]. Our choice of the RF-based tree completion was driven by the availability of the fast B-RF(+) algorithm, which we adopted. Note that our method of forming *X* is heuristic and the appropriateness of a criterion (such as RF) is an empirical question. However, there is no reason to think that other tree completion/resolution criteria could not have worked equally well or better.

From an algorithmic perspective, constrained search can be considered an extension of the phylogenetic placement problem [37–40]. Unlike placement, constrained search also infers the relationship between query genomes and hence is more informative. Like placement, constrained searches can be used to grow existing trees in an automated fashion and regular basis, without the need to redo the analysis from scratch each time. Taking this idea one step further, constrained ASTRAL can perhaps help develop new divide-and-conquer meta-methods that allow ASTRAL to scale to much larger datasets than what it can currently handle.

**Fig. 7:**
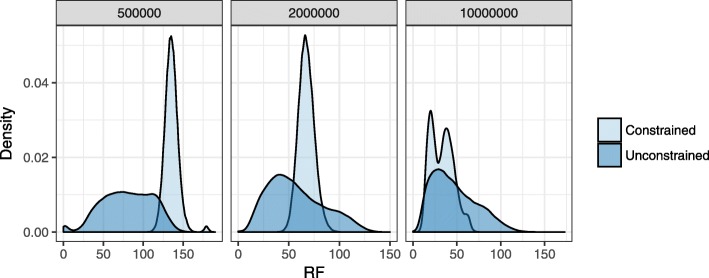
Accuracy of hybrid gene trees. We resolve the ASTRAL-III tree with branches that have support ≤0.99 contracted (as *T*_*b*_) with respect to each gene tree (as *T*_*r*_) using our extended tree completion algorithm and call the resulting tree a hybrid gene tree. Density plots show the error of all 1000 hybrid (Constrained) and original (Unconstrained) gene trees, measured using RF distance to true gene trees (out of 396)

By completing and resolving the constrained species tree using input gene trees, we produce a “hybrid” between individual gene trees and the incomplete/unresolved species tree. We showed these hybrid trees are effective in defining search space but we also wondered about accuracy of these trees. Recall that our estimated gene trees have high estimation error (25% to 47% mean RF). Inspired by tree fixing methods [41], we ask if the hybrid gene trees (which, by construction, have reduced RF to the species tree) are better estimates of the true gene trees than the original gene trees. To answer this question, we estimated unconstrained ASTRAL-III trees, collapsed branches with ≤0.99 support, and used the resulting tree as *T*_*b*_ and each gene tree as *T*_*r*_ as input to the tree completion algorithm. The resulting hybrid trees had mixed accuracy (Fig. 7). With moderate ILS, hybrid gene trees have reduced error compared to original estimated gene trees; here, true gene trees are similar to the species tree (15% mean RF), and reducing distance to the (collapsed) species tree reduces the error. However, with higher ILS, original gene trees are more accurate perhaps because true gene trees are dissimilar to the species tree (69% mean RF) and making gene trees similar to the species tree is not beneficial. Using the hybrid gene trees as input to ASTRAL increases the species tree error in all cases (from 4.3%, 5.6%, 10.2% mean RF to 4.9%, 8.3%, 20.5%, respectively, for moderate, high, and very high ILS and 200 genes).

The constrained ASTRAL search opens the door for several downstream biological analyses. As shown in our reanalyses of the avian dataset, we can now perform hypothesis-driven analyses, as attempted often by systematists. These analyses have the potential to reveal support for some branches that are not recovered in the main ASTRAL tree. The presence of alternative support can also be visualized using tools such as DiscoVista [42]. Moreover, with constrained searches, we can now test if fixing parts of the tree can impact the resolution or support for the other parts. These analyses can help users understand how robust their estimated species trees are. Finally, methods of interrogating the impact of individual genes, such as Partitioned Coalescent Support (PCS) [43] can also benefit from constrained search. Currently, these methods limit themselves to scoring hand-curated trees but they can instead use automatically generated constrained ASTRAL trees.

## Conclusions

We described an algorithm for genome-wide inference of species trees from gene trees while honoring user-provided constraint tree. We have implemented and tested this approach for ASTRAL; however, the same strategy should work for other similar DP methods. Our results showed that the constrained ASTRAL is effective in searching the tree topology space. More interestingly, we showed that constrained search using ASTRAL can help biologists obtain a better understanding of the complexities of genome-wide evolution by revealing support for conflicting resolutions in the species tree.

## Supplementary information

**Additional file 1** Supplementary material.

## Data Availability

Constrained ASTRAL code along with a brief documentation and example for running the code is available on GitHub (https://github.com/maryamrabiee/Constrained-search). Datasets and results are also available on GitHub (https://github.com/maryamrabiee/Constrained-search-data)

## Abbreviations

ASTRAL: Accurate species tree algorithm
DP: Dynamic programming
FN: False negative
FP: False positive
ILS: Incomplete lineage sorting
LCA: Least common ancestor
MP-EST: Maximum Pseudo-likelihood for estimatingsSpecies trees
RF distance: Robinson-foulds distance
