# Supplementary material for: Forcing external constraints on tree inference using ASTRAL
Source: BMC Genomics. 2020 Apr 16;21(Suppl 2):218. doi: 10.1186/s12864-020-6607-z (PMC7160890; doi:10.1186/s12864-020-6607-z)
Supplement: Supplementary file 1 — Additional file 1 Supplementary material. [file 12864_2020_6607_MOESM1_ESM.pdf]

Supplementary material

A Commands and versions used

Running Constrained ASTRAL
Constrained ASTRAL in this paper refers to version 5.6.7 of the code available on:
`https://github.com/maryamrabiee/Constrained-search`
Unconstrained ASTRAL refers to version 5.6.3 of ASTRAL-III available on `https://github.com/smirarab/ASTRAL`
Constrained ASTRAL program were run with following command:

```
java -jar <program> -i <input> -o <output> -e <constrainttree> >
<completedtrees> 2> <log>
```

Contracting Low Support Branches
In order to contract gene tree branches with bootstrap up to a certain threshold we used this command:

```
nw_ed genetree 'i & (b<=$threshold)' o
```

B Supplementary Figures

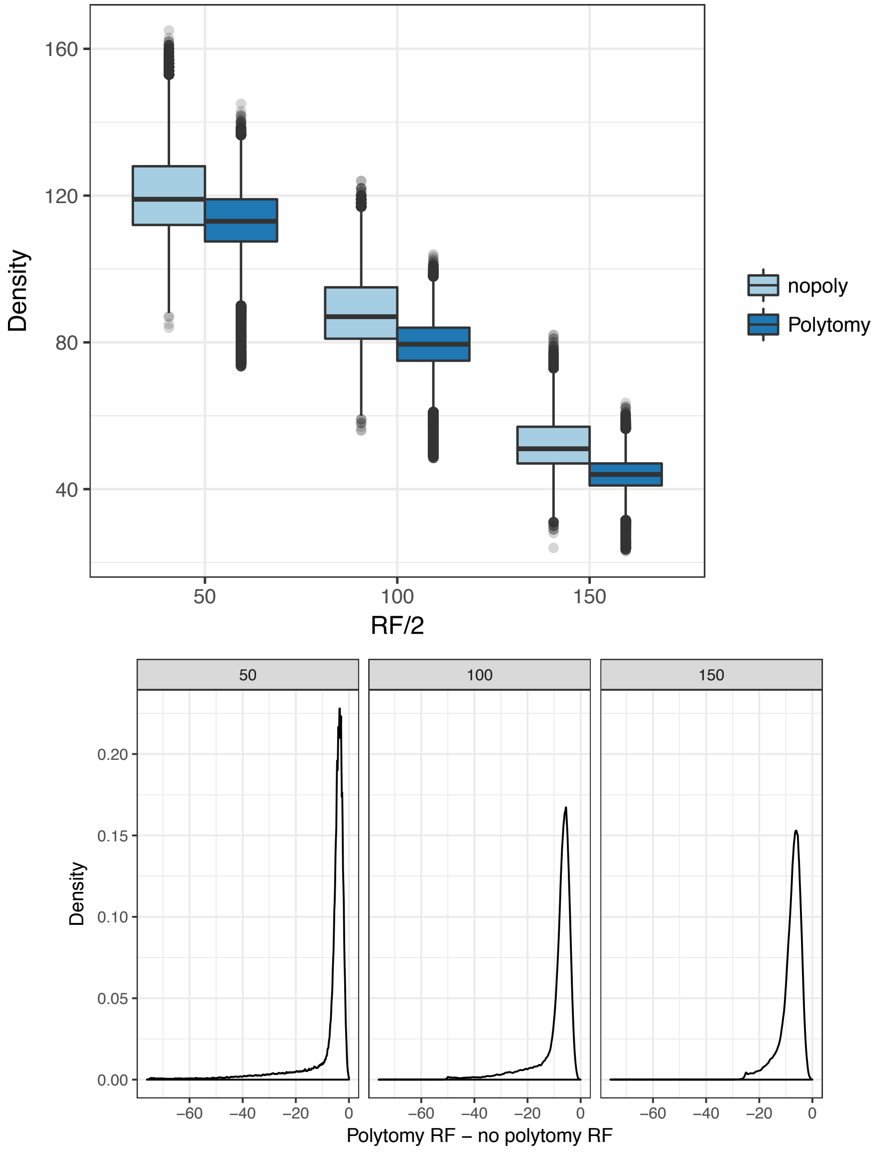

Figure S1 Impact of changes to the B-RF(+) algorithm. Our suggested changes to the B-RF(+) algorithm result in reduced RF distance between completed T_b and T_r. Top: The distribution of RF distance between results of tree completion and T_r with (Polytomy) and without (nopoly) our changes to allow multifurcating trees. Bottom: The distribution of the reduction in RF distance as a result of allowing polytomies in output trees. The drop in RF distance can be as high as 76 edges and is on average 8 branches.

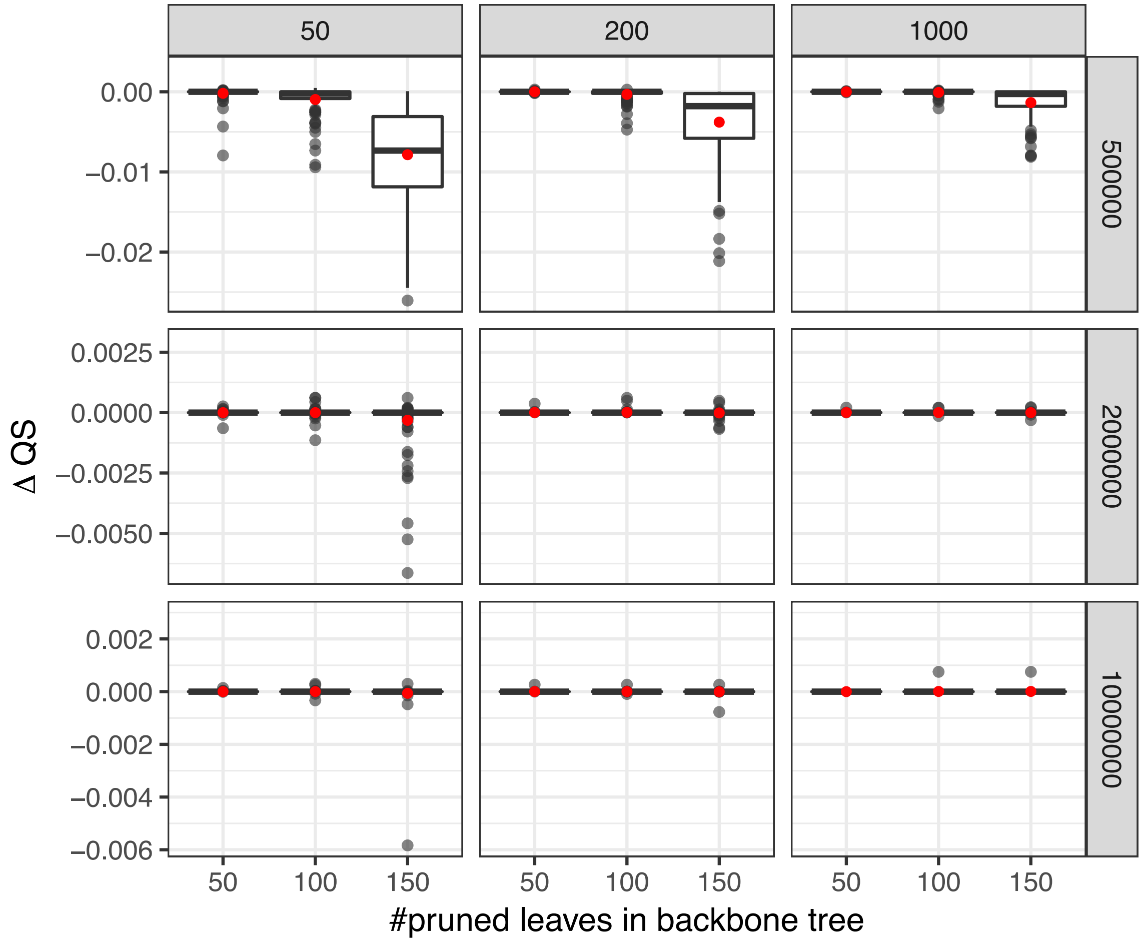

Figure S2 Change in quartet score due to constraints with missing leaves. The quartet score of the constrained search run on the backbone tree with a random set of leaves pruned is compared with unconstrained tree. Positive difference means improvement in the quartet score. There are three different levels of ILS (500K, 1M and 2M generations), varying number of genes (50, 200, 1000) and backbone trees with different sizes.

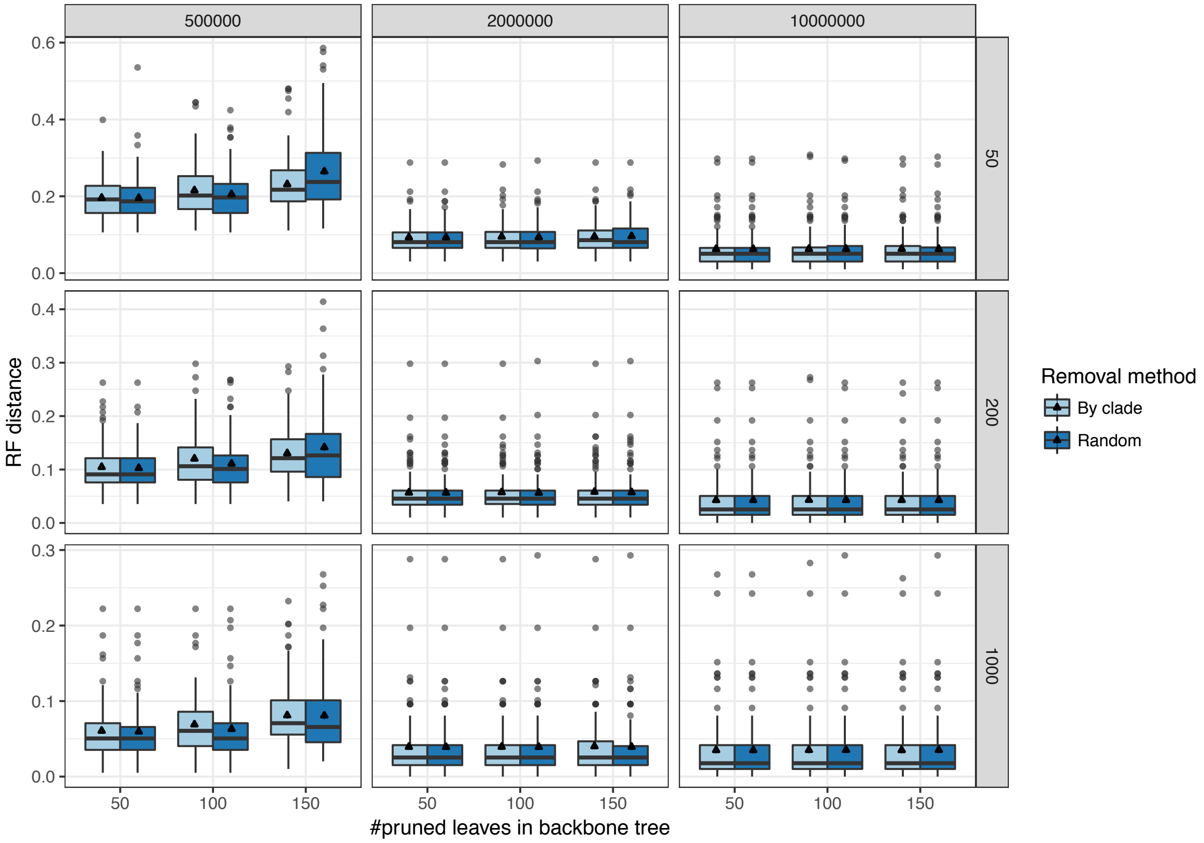

Figure S3 Impact of clade-based removal of species. The two methods for pruning leaves from backbone tree are compared here: random removal and clade-based removal. In the clade-based, for a desired number of pruned leaves, we repeatedly remove a randomly chosen remaining clade in the tree only if pruning the clade does not push us over the limit and continue until the desired size is achieved. Note that the selected nodes could be singletons (leaves) or internal nodes (clades) in the tree. We show Normalized RF distance between inferred constrained ASTRAL tree and the true species tree. There are three different levels of ILS (500K, 1M and 2M generations), varying number of genes (50, 200, 1000) and backbone trees with different sizes.

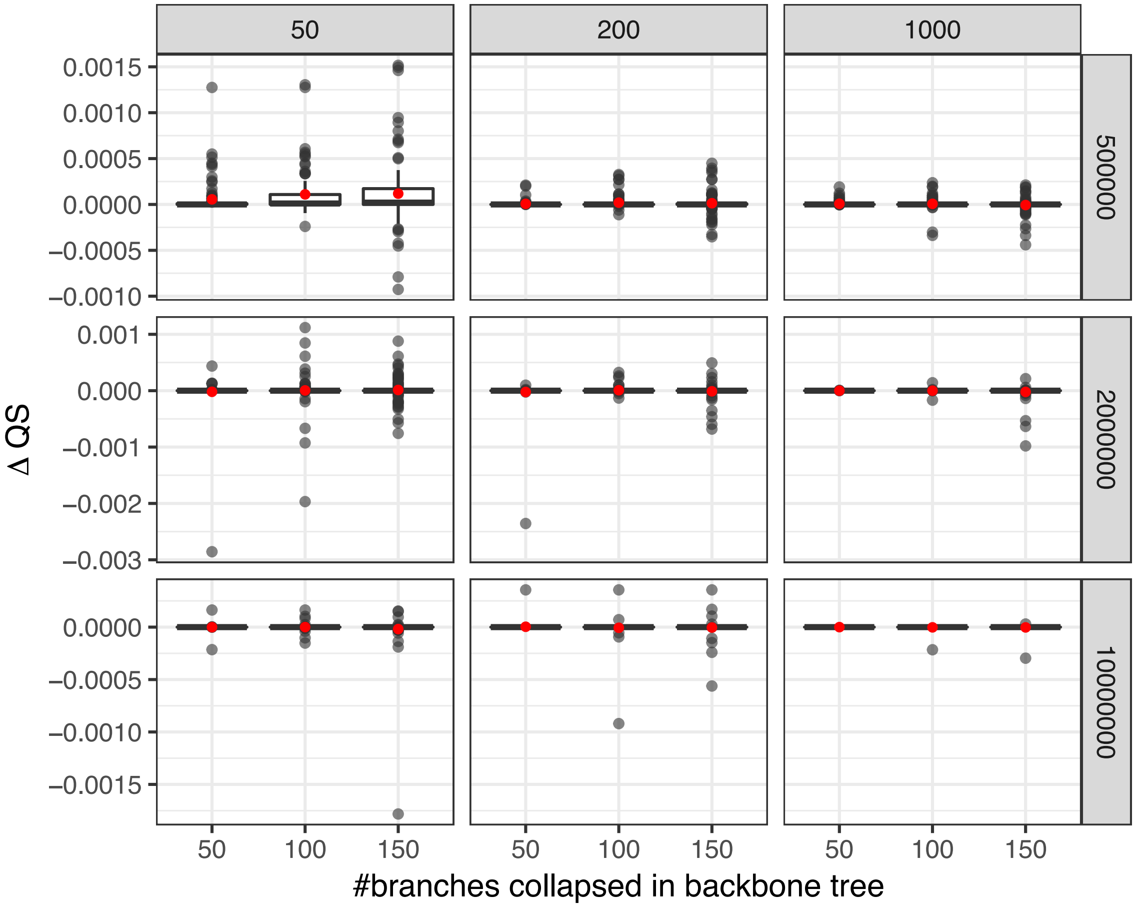

Figure S4 Change in quartet score due to constraints with missing branches. The quartet score of the constrained search run on the backbone tree with randomly collapsed branches is compared with unconstrained tree. Positive difference means improvement in the quartet score. There are three different levels of ILS (500K, 1M and 2M generations), varying number of genes (50, 200, 1000) and backbone trees with different number of branches collapsed. These branches are selected randomly among all $n-1$ internal branches.

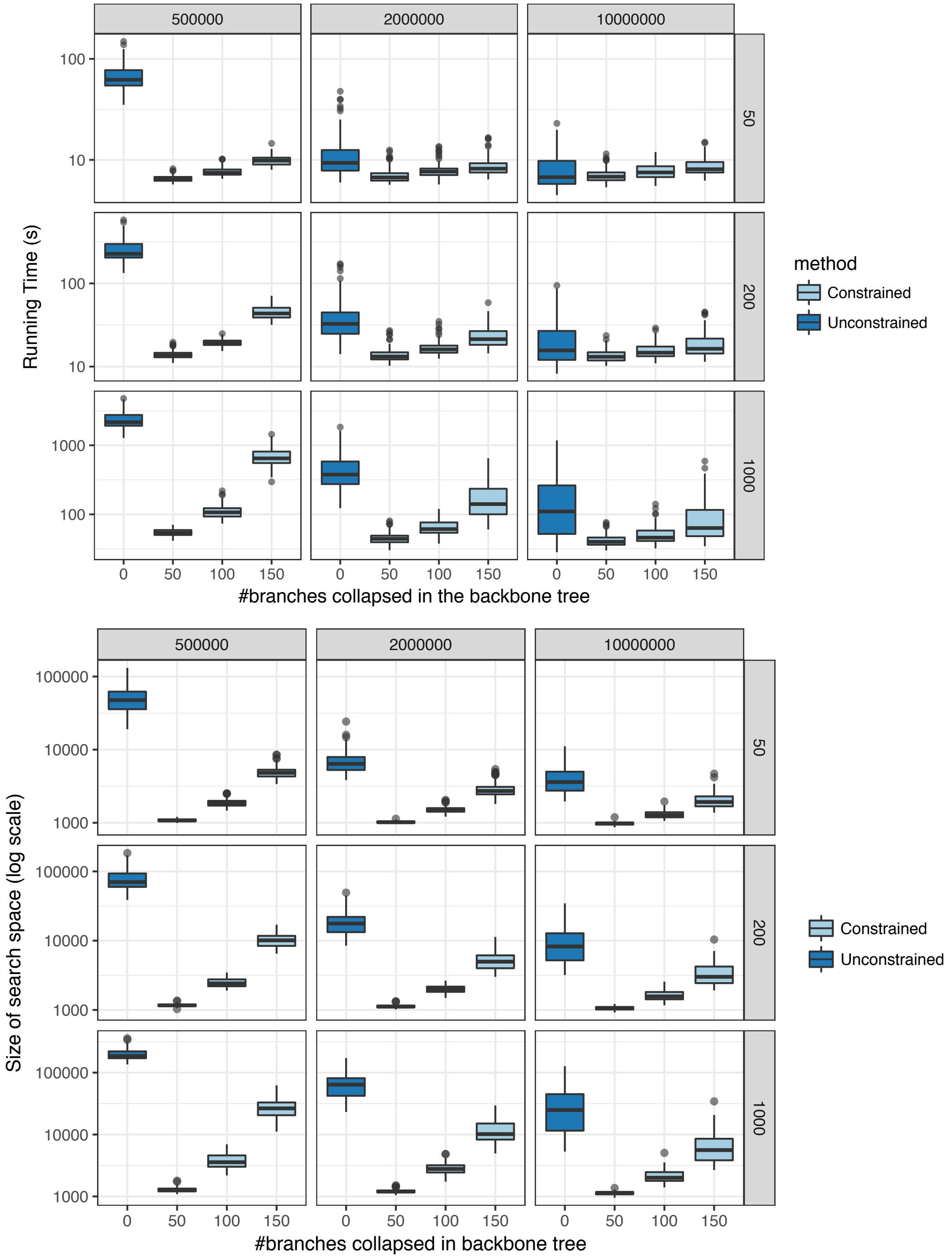

Figure S5 Impact of multifurcating constraint trees on running time and search space. The running time (top) and search space size $|X|$ (bottom) of constrained and unconstrained ASTRAL-III are compared here in log scale. There are three different levels of ILS (500K, 1M and 2M generations), varying number of genes (50, 200, 1000) and backbone trees with different number of branches collapsed. The search space size of constrained and unconstrained ASTRAL-III are compared here in log scale. There are three different levels of ILS (500K, 1M and 2M generations), varying number of genes (50, 200, 1000) and backbone trees with different sizes.
